# Influence of Conversion and Anastomotic Leakage on Survival in Rectal Cancer Surgery; Retrospective Cross-sectional Study

**DOI:** 10.1007/s11605-018-3931-6

**Published:** 2018-09-05

**Authors:** Edgar J. B. Furnée, Tjeerd S. Aukema, Steven J. Oosterling, Wernard A. A. Borstlap, Willem A. Bemelman, Pieter J. Tanis, on behalf of the Dutch Snapshot Research Group

**Affiliations:** 1 Department of Abdominal Surgery, University Medical Center Groningen, Hanzeplein 1, P.O. Box 30001, 9700 RB Groningen, the Netherlands; 2 Department of Surgery, Spaarne Gasthuis, Haarlem, the Netherlands; 3 Department of Surgery, Amsterdam UMC, University of Amsterdam, Amsterdam, the Netherlands

**Keywords:** Rectal cancer, Laparoscopy, Conversion, Anastomosis, Survival

## Abstract

**Background:**

Conversion and anastomotic leakage in colorectal cancer surgery have been suggested to have a negative impact on long-term oncologic outcomes. The aim of this study in a large Dutch national cohort was to analyze the influence of conversion and anastomotic leakage on long-term oncologic outcome in rectal cancer surgery.

**Methods:**

Patients were selected from a retrospective cross-sectional snapshot study. Patients with a benign lesion, distant metastasis, or unknown tumor or metastasis status were excluded. Overall (OS) and disease-free survival (DFS) were compared between laparoscopic, converted, and open surgery as well as between patients with and without anastomotic leakage.

**Results:**

Out of a database of 2095 patients, 638 patients were eligible for inclusion in the laparoscopic, 752 in the open, and 107 in the conversion group. A total of 746 patients met the inclusion criteria and underwent low anterior resection with primary anastomosis, including 106 (14.2%) with anastomotic leakage. OS and DFS were significantly shorter in the conversion compared to the laparoscopic group (*p* = 0.025 and *p* = 0.001, respectively) as well as in anastomotic leakage compared to patients without anastomotic leakage (*p* = 0.002 and *p* = 0.024, respectively). In multivariable analysis, anastomotic leakage was an independent predictor of OS (hazard ratio 2.167, 95% confidence interval 1.322–3.551) and DFS (1.592, 1077–2.353). Conversion was an independent predictor of DFS (1.525, 1.071–2.172), but not of OS.

**Conclusion:**

Technical difficulties during laparoscopic rectal cancer surgery, as reflected by conversion, as well as anastomotic leakage have a negative prognostic impact, underlining the need to improve both aspects in rectal cancer surgery.

## Introduction

Total mesorectal excision (TME), being the cornerstone of rectal cancer treatment, has gradually evolved in the past decade from open surgery to a laparoscopic approach, as it has shown advantageous short-term outcomes and a lower postoperative complication rate, including less pain, improved recovery time, and less blood loss.^1–3^ Its oncologic safety and equivalence to open surgery has been demonstrated in a number of randomized clinical trials.^4–9^ However, TME surgery, both open and laparoscopic, is still associated with considerable morbidity. Both intra-operative and postoperative complications have been associated with shorter overall survival and unfavorable oncologic outcomes,^10,11^ although some studies failed to show a direct relationship.^12,13^ Due to the complex nature of the procedure, conversion from laparoscopic to open surgery is still reported in up to 30% of cases.^14^

Subgroup analysis in the CLASSIC trial has suggested an inferior overall survival in converted patients compared to patients in whom laparoscopic resection was completed successfully, and even worse outcomes compared to primary open resection as well.^6^ In addition, several cohort studies have been published reporting the long-term oncologic outcome in patients who were converted during laparoscopic colorectal cancer surgery.^14^ Although some studies have shown significant differences in long-term oncologic outcome between the laparoscopic and converted patients, other studies have failed to confirm these differences. Additionally, the vast majority of these studies included both colon and rectal cancer patients and did not report the outcomes for colon and rectal cancer patients separately. The few studies that merely reported on rectal cancer patients only included a small patient population. Due to these drawbacks, it is unclear from the current literature what the real influence of conversion on the long-term outcome in rectal cancer patients is.

Furthermore, in the direct postoperative phase, anastomotic leakage (AL) with a reported incidence ranging from 4 to 19% remains a major source of morbidity and also mortality. A comprehensive meta-analysis showed the negative impact of AL on oncologic outcome,^10^ although other studies did not.^12,13^ In addition, most of these studies provide retrospective mono-center cohort series with variable definitions of AL.

The aim of this study in a large Dutch national cohort of patients with rectal cancer was to analyze the influence of conversion in the subgroup of patients who were intentionally treated by laparoscopy as well as the influence of AL in the subgroup of patients who underwent low anterior resection with primary anastomosis on the long-term oncologic outcome.

## Material and Methods

### Snapshot Design

A resident-led, retrospective cross-sectional snapshot study, a method first described by Bhangu et al.,^15^ was conducted in 71 hospitals in the Netherlands. This included all consecutive patients who underwent surgery for primary rectal cancer from January to December 2011. It was executed as collaborative research under the name of the Dutch Snapshot Research Group (DSRG), in collaboration with the Dutch Surgical Colorectal Audit (DSCA).

### Ethics

The Medical Ethical Committee of the Academic Medical Centre in Amsterdam, the Netherlands, reviewed and approved the study design and judged that no informed consent from the included patients was necessary considering the observational study design with no additional burden for the patient.

### Data Extraction

The methodology of this snapshot study has been described elaborately in the first publication of the DSRG.^16^ Briefly, all patients who had resection for rectal cancer in 2011 were identified from the DSCA. Existing data from the DSCA were completed by the snapshot study, including additional data on diagnostic and treatment characteristics and long-term surgical and oncologic outcomes. Every participating hospital had one or two surgical residents who, supervised by a surgeon, were responsible for collection of additional data that subsequently could be entered into a web-based tool which was specifically developed and controlled on privacy regulations.

### Patients

For the current analysis, all patients with invasive rectal cancer were selected from the database. Patients who underwent resection for a benign lesion, i.e., polyp (T0 or Tis), as well as patients with distant metastasis or patients in whom the tumor (T) or metastasis (M) status was unknown, were excluded.

For analysis with regard to surgical approach, patients were subdivided into three groups: patients in whom resection was successfully completed by laparoscopy (laparoscopic group), patients who were converted to open surgery after initial laparoscopic approach (conversion group), and patients who were primarily operated on by an open approach (open group). Long-term oncologic outcome, i.e., overall (OS) and disease-free survival (DFS) as well as local and distant recurrence, in the conversion group was compared to the successful laparoscopic as well as to the primary open group.

For analysis of the AL group, only patients who underwent low anterior resection (LAR) with primary anastomosis with or without diverting ileo- or colostomy were included. Patients who had LAR after previous transanal endoscopic microsurgery (TEM) were excluded. In the included group of patients, long-term oncologic outcome in patients who developed AL during the postoperative period (within 30 days from surgery) was compared to patients who did not have AL. In addition, OS and DFS were also compared between patients with and without protective ileo- or colostomy in the subgroup of patients with AL.

LAR was defined as total or partial mesorectal excision with the formation of a primary colorectal or colo-anal anastomosis. AL was defined as the presence of contrast extravasation, presacral fluid collection, or presacral sinus on imaging studies requiring surgical, radiological, or endoscopic intervention.

### Statistical Analysis

Data were analyzed using SPSS 17.0 for Windows (SPSS Inc., Chicago, IL, USA). Continuous values were expressed as mean ± standard deviation (SD) or median (range), depending on whether the data were normally distributed or not, respectively. Categorical data were reported as frequencies with percentages. The *t* test for independent samples was used for statistical analysis of continuous values between groups. Statistical analysis of categorical values between groups was performed by using the Pearson chi-square test or Fisher’s exact test, where appropriate. The Kaplan-Meier method was used to report OS and DFS, and the log-rank test for statistical analysis between groups. Differences between groups were considered statistically significant with *p* value less than 0.05.

Uni- and multivariable Cox regression analysis was performed to identify independent predictive variables for OS and DFS in the group of patients in whom the initial approach was by laparoscopy (independently whether conversion was necessary or not, i.e., patients from the laparoscopic and from the conversion group were included and patients from the open group were excluded for this analysis) as well as in the group of patients who had LAR with primary anastomosis. First, univariable analysis was performed in both separate groups for OS as well as DFS by the Kaplan-Meier method and differences between groups were analyzed using the log-rank test. The variables tested were gender, age, body mass index, ASA score, tumor stage, nodal status, positive resection margin, multi-visceral resection, intra-operative and postoperative complications, and postoperative transfusion needed. In the laparoscopic group, conversion was also added as variable and AL was added as variable in the group of patients with LAR and primary anastomosis. Variables with *p* < 0.10 in univariable analysis were entered together into a multivariable analysis performed by Cox regression analyses. Variables with *p* < 0.05 in multivariable analysis were considered to be significant predictors of survival. The hazard ratio and 95% confidence interval were presented for every predictive variable in multivariable analysis.

## Results

The snapshot database contained a total of 2095 patients. Distant metastases were present in 163 patients (7.8%), and in 177 patients (8.4%), M-status was unknown (Mx). Tumor status was unknown (Tx) in 45 patients (2.1%) and 133 patients (6.3%) had a benign rectal lesion. In the latter two groups, a total of 25 patients had distant metastasis or unknown M-status. In 105 patients, T and/or M-status were not reported in the database. All these patients were excluded for analysis in the present study.

### Conversion

For analysis with regard to surgical approach, 638 patients were available for inclusion in the laparoscopic group, 752 in the open group, and 107 in the conversion group. Reasons for conversion were insufficient abdominal access in 82 patients (76.6%), tumor-related factors in 15 (14.0%), and intra-operative complication in eight patients (7.5%). The reason for conversion was not reported in the remaining two patients. The baseline characteristics of the three separate groups are shown in Table 1. Body mass index was significantly higher in the conversion group compared to both other groups. In addition, more ASA III/IV patients and more patients with T4 tumor were included in the conversion group compared to the laparoscopic group. There was no significant difference between the groups for the other baseline characteristics. With regard to intra- and postoperative data, there was a significant difference in the type of rectal resection between the laparoscopic and conversion group and there were more multi-visceral resections in the latter group (Table 2). In addition, there were more intra-operative complications, more postoperative blood transfusions needed, and longer hospital stay in the conversion compared to the laparoscopic group, whereas postoperative morbidity and mortality was not different between both groups. Comparison of the open and conversion group showed a significant difference in the type of ostomy and in addition, significantly more postoperative blood transfusions were needed in the conversion group (Table 2).

**Table 1:** Baseline characteristics

	Surgical approach					Postoperative morbidity		
	Laparoscopic group (*n* = 638)	Open group (*n* = 752)	Conversion group (*n* = 107)	*p* value (lapsc vs. conversion)	*p* value (open vs. conversion)	Anastomotic leakage group (*n* = 106)	No anastomotic leakage group (*n* = 640)	*p* value
Male gender (%)	395 (61.9)	482 (64.1)	74 (69.2)	0.151	0.184	68 (64.2)	409 (63.9)	0.977
Age (years)	66.4 (11.0)	67.7 (11.3)	68.5 (11.0)	0.066	0.487	63.0 (10.0)	64.9 (10.6)	0.220
Body mass index (kg/m^2^)	25.6 (3.8)	26.1 (4.2)	27.3 (4.4)	< 0.001	0.005	25.7 (4.0)	26.1 (3.8)	0.532
Co-morbidities (%)	433 (67.9)	533 (70.9)	78 (72.9)	0.300	0.387	69 (65.1)	413 (64.5)	0.911
ASA score III/IV (%)	78 (12.2)	148 (19.7)	27 (25.2)	0.002	0.614	14 (13.2)	76 (11.9)	0.696
Previous abdominal surgery (%)	165 (25.9)	246 (32.7)	30 (28.0)	0.603	0.354	26 (24.5)	162 (25.3)	0.856
Emergency surgery (%)	5 (0.9)	15 (2.0)	2 (1.9)	0.342	0.656	0 (0)	7 (1.1)	0.672
Second primary colorectal cancer (%)	7 (1.1)	29 (3.9)	2 (1.9)	0.296	0.627	2 (1.9)	14 (2.2)	0.129
Neo-adjuvant treatment								
- Short course RT short interval (%)	335 (52.5)	282 (37.5)	58 (54.2)	0.114		57 (53.8)	319 (49.8)	
- Short course RT long interval (%)	43 (6.7)	41 (5.5)	3 (2.8)			5 (4.7)	27 (4.2)	0.638
- Long course chemo-radiotherapy (%)	177 (27.7)	245 (32.6)	25 (23.4)		0.189	33 (31.1)	160 (25.3)	
- Long course RT alone	20 (3.1)	28 (3.7)	2 (1.9)			2 (1.9)	17 (2.7)	
Systemic chemotherapy (%)	6 (0.9)	24 (3.2)	1 (0.9)			2 (1.9)	18 (2.8)	
TNM-classification								
- pT4 (%)	13 (2.0)	51 (6.8)	8 (7.5)	0.002	0.573	2 (1.9)	16 (2.5)	0.703
- pN1–2 (%)	220 (34.5)	234 (31.1)	45 (42.1)	0.373	0.140	37 (34.9)	223 (34.8)	0.984
Positive resection margin	17 (2.7)	34 (4.5)	3 (2.8)	0.907	0.649	2 (1.9)	18 (2.8)	0.583
Time to follow-up (months)	41 (3–54)	42 (1–55)	42 (4–54)	0.781	0.159	41 (1–52)	42 (1–54)	0.405

Values are reported as mean (SD) or median (range)

*RT*, radiotherapy; *ns*, not significant; *lapsc*, laparoscopic group

**Table 2:** Intra- and postoperative data

	Surgical approach					Postoperative morbidity		
	Laparoscopic group (*n* = 638)	Open group (*n* = 752)	Conversion group (*n* = 107)	*p* value (lapsc vs. conversion)	*p* value (open vs. conversion)	Anastomotic leakage group (*n* = 106)	Non-anastomotic leakage group (*n* = 640)	*p* value
Type of operative procedure								
- Low anterior resection (%)	361 (56.6)	330 (43.9)	55 (51.4)	0.002	0.091	NA	NA	
- Abdomino-perineal resection (%)	191 (29.9)	251 (33.4)	22 (20.6)			NA	NA	
- Hartmann’s procedure (%)	81 (12.7)	161 (21.4)	30 (28.0)			NA	NA	
- Proctocolectomy (%)	5 (0.8)	10 (1.3)	0 (0)			NA	NA	
Multi-visceral resection (%)	24 (3.8)	71 (9.4)	10 (9.3)	< 0.001	0.525	7 (6.6)	19 (3.0)	0.055
Type of stoma								
- Diverting loop ileostomy (%)	224 (35.1)	180 (23.9)	42 (39.3)	0.243	0.009	57 (53.8)	399 (62.3)	0.167
- Diverting loop colostomy (%)	6 (0.9)	44 (5.9)	1 (0.9)			6 (5.7)	45 (7.0)	
- End colostomy (%)	273 (42.8)	407 (54.1)	52 (48.6)			NA	NA	
- End ileostomy (%)	10 (1.6)	16 (2.1)	2 (1.9)			NA	NA	
Total intra-operative complications (%)	12 (1.9)	24 (3.2)	8 (7.5)	0.001	0.088	2 (1.9)	11 (1.7)	0.130
- Bleeding requiring transfusion (%)	2 (0.3)	11 (1.5)	5 (4.7)			0 (0)	7 (1.1)	
- Bowel injury (%)	2 (0.3)	8 (1.1)	1 (0.9)			2 (1.9)	2 (0.3)	
- Ureter injury (%)	6 (0.9)	4 (0.5)	2 (1.9)			0 (0)	2 (0.3)	
- Other (%)	2 (0.3)	1 (0.1)	0 (0)			0 (0)	0 (0)	
Postoperative complication (%)	213 (33.4)	313 (41.6)	43 (40.2)	0.341	0.892	NA	205 (32.0)	NA
Anastomotic leakage (%)*	48 (13.3)	52 (15.8)	4 (7.3)	0.275	0.145	NA	NA	NA
Postoperative transfusion needed (%)	46 (7.2)	147 (19.5)	30 (28.0)	< 0.001	0.038	19 (17.9)	51 (8.0)	0.001
Mortality (30 days) (%)	14 (2.2)	23 (3.1)	2 (1.9)	0.827	0.759	4 (3.8)	13 (2.0)	0.250
Hospital stay (days)	7 (2–191)	9 (2–235)	10.5 (2–114)	0.003	0.062	13 (3–120)	8 (2–235)	< 0.001

Values are reported as median (range)

*ns*, not significant; *lapsc*, laparoscopic group

*Percentage in patients who underwent low anterior resection with primary anastomosis

### Anastomotic Leakage

From the snapshot database, a total of 998 patients underwent LAR with primary anastomosis with or without diverting ostomy. After exclusion of patients who met the exclusion criteria as described, 746 patients, including 106 (14.2%) with AL, were available for analysis in the present study. There were no statistically significant differences between both groups with regard to baseline characteristics (Table 1). During the postoperative period, more blood transfusions were needed in the group of patients with AL and hospital stay was significantly longer in this group (Table 2).

### Long-term Oncologic Outcome

**Fig. 1:**
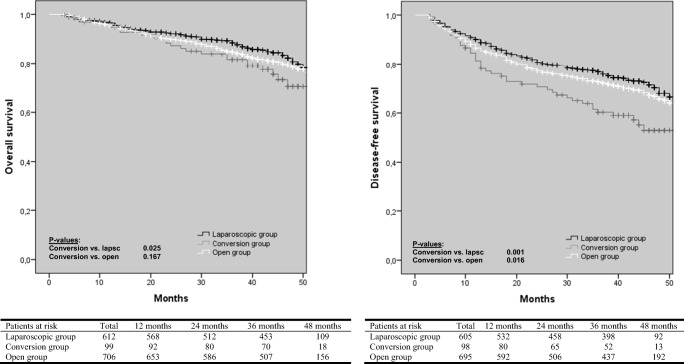
Kaplan-Meier curves of overall and disease-free survival according to surgical approach

**Fig. 2:**
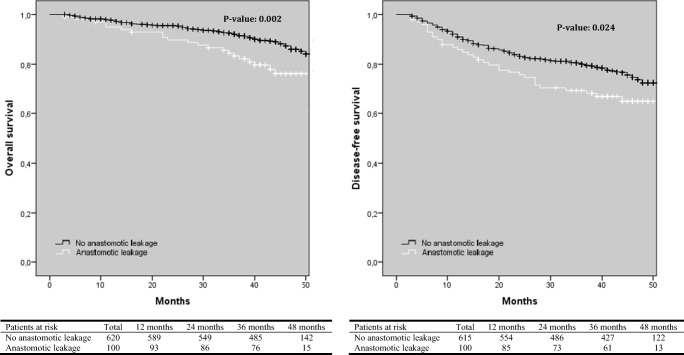
Kaplan-Meier curve of overall and disease-free survival in patients with or without anastomotic after LAR and primary anastomosis

**Fig. 3:**
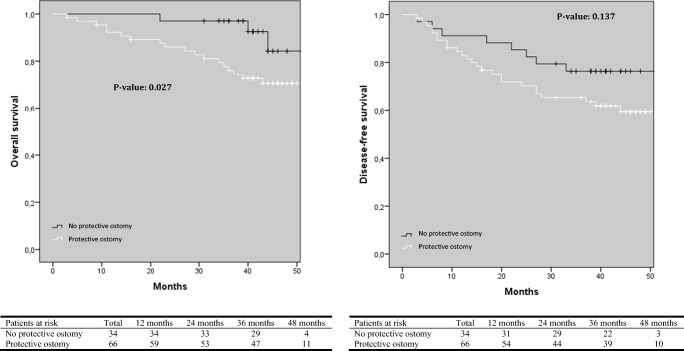
Kaplan-Meier curve of overall and disease-free survival in patients with anastomotic leakage with or without a protective ileo- or colostomy

Time to long-term follow-up in every group is shown in Table 1. With regard to OS, the laparoscopic group had a significantly better OS compared to the conversion group (*p* = 0.025), although a significant difference between the primary open and conversion group was not found (Fig. 1). With regard to DFS, there was a significant difference between the conversion and laparoscopic group in favor of the latter one (*p* = 0.001, Fig. 1). In addition, DFS was also significantly worse after conversion compared to primary open surgery (*p* = 0.016). OS and DFS were significantly worse in patients with AL compared to patients without AL (Fig. 2). In the group of patients with AL, the presence of a diverting ileo- or colostomy had a negative effect on OS, but there was no significant difference with regard to DFS (Fig. 3).

There was no significant difference in local recurrence rate, neither between the laparoscopic and open vs. conversion group (*n* = 32 (5.2%), *n* = 43 (6.1%), and *n* = 8 (8.1%), respectively) nor between patients with or without AL (*n* = 4 (3.8%) and *n* = 21 (3.3%), respectively). However, there was a significant difference in distant metastasis rate between the laparoscopic and conversion group (*n* = 110 (18.0%) vs. *n* = 31 (31.2%), *p* = 0.004). Differences between both groups for specific locations of distant metastasis, i.e., liver, pulmonary, para-aortal lymph node, bone, or peritoneal, were not found. There was no significant difference in distant recurrence rate found between patients with or without AL (*n* = 24 (22.9%) and *n* = 116 (18.3%), *p* = 0.287).

### Uni- and Multivariable Analysis

The results of uni- and multivariable analysis for OS and DFS in the laparoscopic group are shown in Table 3. For OS, 10 variables found in univariable analysis were entered into multivariable analysis. Finally, five variables were identified as significant predictors of OS in the laparoscopic group, including age > 60 years, ASA score, nodal status, positive resection margin, and postoperative transfusion needed. For DFS, six out of nine variables remained as significant predictors in the laparoscopic group: ASA score, nodal status, positive resection margin, multi-visceral resection, conversion, and postoperative complications.

**Table 3:** Results of uni-/multivariable analysis for overall and disease-free survival in laparoscopic group

	Overall survival						Disease-free survival					
Variable	Total patients	Overall survival	Univariable *p* value	Multivariable *p* value	Hazard ratio	95% confidence interval	Total patients	Disease-free survival	Univariable *p* value	Multivariable *p* value	Hazard ratio	95% confidence interval
Gender												
- Male	446	365 (81.8%)	0.044	NS			441	302 (68.5%)	0.032	NS		
- Female	265	232 (87.5%)					262	200 (76.3%)				
Age > 60 years												
- < 60 years	204	186 (91.2%)	< 0.001	0.004	2.164	1.271–3.686	200	144 (72.0%)	0.563	–		
- > 60 years	507	411 (81.1%)					503	358 (71.2%)				
Body mass index												
- < 30 kg/m^2^	588	486 (82.7%)	0.112	–			581	410 (70.6%)	0.446	–		
- > 30 kg/m^2^	91	81 (89.0%)					90	67 (74.4%)				
ASA score												
- I/II	619	534 (86.3%)	< 0.001	0.003	2.012	1.261–3.210	611	450 (73.6%)	< 0.001	0.001	1.799	1.255–2.580
- III/IV	92	63 (68.5%)					92	52 (56.5%)				
Tumor stage												
- pT1–3	694	585 (84.3%)	0.132	–			686	494 (72.0%)	0.014	NS		
- pT4	17	12 (70.6%)					17	8 (47.1%)				
Nodal status												
- pN0	444	388 (87.4%)	0.002	0.002	1.823	1.242–2.675	441	353 (80.0%)	< 0.001	< 0.001	2.323	1.742–3.096
- pN1–2	252	198 (78.6%)					247	141 (57.1%)				
Positive resection margin												
- R0	687	580 (84.4%)	0.005	0.012	2.735	1.244–6.014	680	491 (72.2%)	< 0.001	0.009	2.391	1.242–4.601
- R1–2	19	12 (63.2%)					17	6 (35.3%)				
Multi-visceral resection												
- Yes	30	21 (70.0%)	0.042	NS			30	15 (50.0%)	0.004	0.024	1.845	1.085–3.137
- No	678	574 (84.7%)					670	485 (72.4%)				
Intra-operative complication												
- Yes	16	12 (75.0%)	0.088	NS			16	11 (68.8%)	0.424	–		
- No	682	577 (84.6%)					674	484 (71.8%)				
Conversion												
- Yes	99	75 (75.8%)	0.025	NS			98	57 (58.2%)	0.001	0.019	1.525	1.071–2.172
- No	612	522 (85.3%)					605	445 (73.6%)				
Postoperative complication												
- Yes	236	190 (80.5%)	0.090	NS			232	152 (65.5%)	0.013	0.026	1.393	1.040–1.865
- No	471	403 (85.6%)					467	346 (74.1%)				
Postoperative transfusion needed												
- Yes	67	46 (68.7%)	< 0.001	0.002	2.240	1.360–3.690	66	38 (57.6%)	0.001	NS		
- No	623	533 (85.6%)					616	450 (73.1%)				

For patients who underwent LAR with primary anastomosis, results of uni- and multivariable analysis for OS and DFS are shown in Table 4. For OS, age > 60 years, nodal status, positive resection margin, AL, and postoperative transfusion needed were independent predictors. Four variables, including nodal status, positive resection margin, AL, and postoperative transfusion needed, were significant predictors of DFS in this group.

**Table 4:** Results of uni-/multivariable analysis for overall and disease-free survival in patients with LAR and primary anastomosis

Overall survival							Disease-free survival					
Variable	Total patients	Overall survival	Univariable *p* value	Multivariable *p* value	Hazard ratio	95% confidence interval	Total patients	Disease-free survival	Univariable *p* value	Multivariable *p* value	Hazard ratio	95% confidence interval
Gender												
- Male	458	394 (86.0%)	0.146	–			456	338 (74.1%)	0.276	–		
- Female	261	234 (89.7%)					258	200 (77.5%)				
Age > 60 years												
- < 60 years	240	218 (90.8%)	0.021	0.027	1.734	1.063–2.826	236	178 (75.4%)	0.805	–		
- > 60 years	480	411 (85.6%)					479	361 (75.4%)				
Body mass index												
- < 30 kg/m^2^	282	244 (86.5%)	0.700	–			593	446 (75.2%)	0.831	–		
- > 30 kg/m^2^	409	361 (88.3%)					94	72 (76.6%)				
ASA score												
- I/II	636	562 (88.4%)	0.012	NS			631	482 (76.4%)	0.059	NS		
- III/IV	84	67 (79.8%)					84	57 (67.9%)				
Tumor stage												
- pT1–3	703	615 (87.5%)	0.365	–			698	531 (76.1%)	0.001	NS		
- pT4	17	14 (82.4%)					17	8 (47.1%)				
Nodal status												
- pN0	464	426 (91.8%)	< 0.001	< 0.001	2.840	1.849–4.361	463	388 (83.8%)	< 0.001	< 0.001	2.980	2.188–4.060
- pN1–2	246	195 (79.3%)					242	144 (59.5%)				
Positive resection margin												
- R0	695	611 (87.9%)	< 0.001	0.006	3.084	1.376–6.916	690	525 (76.1%)	< 0.001	0.001	2.997	1.608–5.588
- R1–2	19	12 (63.2%)					19	8 (42.1%)				
Multi-visceral resection												
- Yes	25	18 (72.0%)	0.037	NS			25	12 (48.0%)	0.001	NS		
- No	674	590 (87.5%)					669	508 (75.9%)				
Intra-operative complication												
- Yes	13	10 (76.9%)	0.210	–			13	9 (69.2%)	0.483	–		
- No	688	603 (87.6%)					683	514 (75.3%)				
Anastomotic leakage												
- Yes	100	78 (78.0%)	0.002	0.002	2.167	1.322–3.551	100	67 (67.0%)	0.024	0.020	1.592	1.077–2.353
- No	620	551 (88.9%)					615	472 (76.7%)				
Postoperative complication												
- Yes	271	231 (85.2%)	0.164	–			268	197 (73.5%)	0.245	–		
- No	447	396 (88.6%)					445	340 (76.4%)				
Postoperative transfusion needed												
- Yes	63	44 (69.8%)	< 0.001	< 0.001	2.760	1.629–4.677	63	35 (55.6%)	< 0.001	< 0.001	2.159	1.427–3.267
- No	634	564 (89.0%)					629	487 (77.4%)				

## Discussion

The results of this large national cohort study showed that patients requiring conversion in laparoscopic rectal cancer surgery, compared to patients in whom resection was successfully completed by laparoscopy, had a significantly worse OS and DFS. However, conversion was only an independent predictor of DFS and not of OS after correction for confounders. Postoperative AL after rectal resection with primary anastomosis also had a negative impact on OS and DFS, and AL was identified as independent predictor of worse OS as well as DFS.

Although multiple studies have shown a relationship between AL and disease recurrence as well as OS,^17–21^ other studies have not found an adverse effect on oncologic outcome as was demonstrated in the present patient cohort.^12,22^ This might be depending on the various definitions of AL used in the different studies. In the current study, patients with a proven leak on imaging requiring a radiological or surgical intervention were included in the leak group. In the case of successful conservative treatment, patients were included in the non-leakage group. The results of our cross-sectional snapshot study are in accordance with several earlier reports that were comprehensively evaluated in a meta-analysis by Mirnezami et al.^10^ Their results show a distinct negative prognostic impact of AL on local recurrence and long-term survival in rectal cancer patients. However, many studies included in this meta-analysis are outdated and provide scarce or no information on neo-adjuvant therapies or operation techniques, including TME. More recently, Lu et al. included 11 cohort studies in their meta-analysis and concluded that AL following rectal cancer resections using TME had an adverse impact on cancer specific mortality and the rate of local recurrence.^23^ Our study provides detailed pre- and intra-operative information combined with long-term follow-up, exclusively focusing on rectal cancer. This enabled us to correct for several potential confounding factors, such as BMI, ASA classification, intra-operative complications, and tumor stage. The underlying pathophysiological mechanism remains largely conceptual. Several experimental models have demonstrated that inflammation provides a micro-environment that facilitates adhesion and outgrowth of exfoliate tumor cells or micro-metastases.^24^ In addition, prolonged sepsis and inflammatory responses have been shown to be independent predictors of poor survival possibly caused by a less effective immune response against circulating tumor cells.^25^

The negative impact of conversion on survival can be explained by the significant difference in several factors between the laparoscopic and conversion group. In the conversion group in the current study, body mass index was significantly higher and more patients had a higher ASA score. In addition, more patients in the conversion group had a T4 tumor and consequently, more multi-visceral resections were performed. A higher BMI and tumor stage in the conversion group was also reported by others,^26–32^ and in addition, Biondi et al.^33^ found that BMI and a higher tumor stage, in addition to tumor size, were independent predictors of conversion. All these factors might have a negative impact on oncologic outcome and survival. In addition, the blood transfusion rate was also significantly higher in the conversion group. It has previously been suggested that postoperative blood transfusion might have a negative impact on oncologic outcomes due to the release of inflammatory mediators in response to blood transfusion.^34^ Since conversion was not identified as an independent predictor of OS in multivariable analysis, it is plausible that the difference in OS between the successful laparoscopic and conversion group is caused by the overrepresentation of the abovementioned factors in the latter group. So, we cannot conclude that conversion itself negatively influenced OS. However, conversion was found to be a significant predictor of DFS, independent of the other factors which were also included in multivariable analysis. This suggests that, in addition to the negative factors that were overrepresented in the conversion group, conversion itself might also have a negative individual impact on DFS. The worse DFS in the conversion group as well as the identification of conversion as independent predictor of DFS might be explained by a significantly higher distant metastasis rate in the conversion compared to the successful laparoscopic group. It has been suggested that conversion, due to the more extensive tissue dissection, leads to an inflammatory response that compromises the immune system which has a negative impact on oncologic outcome with a higher risk of distant metastasis as a consequence.^35^

Multivariable analysis in the laparoscopic group in the current study identified five independent predictors for OS (age, ASA score, nodal status, positive resection margin, postoperative transfusion) and six for DFS (ASA score, nodal status, positive resection margin, multi-visceral resection, conversion, postoperative complications). Allaix et al.^26^ also performed a multivariable analysis in a cohort of patients who underwent laparoscopic colorectal cancer resection. They found tumor stage and lymph node ratio as independent predictors of survival. Franko et al.^36^ identified age and tumor stage as independent predictors and Li et al.^37^ found, in a cohort of colon cancer patients, tumor stage and poor differentiation as independent predictors of OS and tumor stage, poor differentiation, AL, and no adjuvant chemotherapy as predictors of DFS. In none of these studies, conversion was identified as predictor of survival as we did for DFS.

Previous studies also comparing the long-term oncologic outcome in successful laparoscopy vs. conversion in colorectal cancer surgery were recently summarized in a review.^14^ There were three studies showing a significant difference in OS^26,31,33^ and five in DFS,^26,30,33,38,39^ all in favor of the successful laparoscopic group. However, all of these studies included a heterogeneous group of patients with colon as well as rectal cancer patients. A total of four studies included in this review only reported on rectal cancer patients and in none of these a significant difference in survival was found.^27,29,40,41^ This is probably due to the relatively small number of patients included in those studies as no more than 300 patients were included in the largest one. So, although the study design of the current snapshot study is retrospective, the relatively large patient population increases statistical power. Hereby, we were, for the first time, able to identify a negative impact of conversion on DFS in laparoscopic rectal cancer surgery.

In addition to the retrospective study design, the lack of data on the number, volume, and level of skills training of the individual surgeons involved was another limitation of the current study as variability in expertise might be related to AL and conversion rate. However, a certain level of expertise and a minimal annual volume of 20 rectal resections is required in the Netherlands to be able to perform rectal cancer surgery guaranteeing the quality of surgery. In addition, the hospital volume was the subject of another analysis with the complete cohort of 2095 patients in this snapshot study and no significant impact of hospital volume on rectal cancer surgery outcome was found in that analysis.^42^ Finally, the influence of adjuvant chemotherapy could not be analyzed in the current study, because adjuvant chemotherapy in rectal cancer is not recommended according to the national guideline in the Netherlands.^43,44^

## Conclusion

Technical difficulties during laparoscopic rectal cancer surgery, as reflected by conversion, have an independent impact on long-term outcome, i.e., on DFS, after nationwide implementation of the technique. Also, anastomotic leakage has a prognostic impact, underlining the need to improve both aspects of rectal cancer surgery.
